# Registration and local production of essential medicines in Uganda

**DOI:** 10.1186/s40545-020-00234-2

**Published:** 2020-08-11

**Authors:** Petra Brhlikova, Karen Maigetter, Jude Murison, Amon G. Agaba, Jonans Tusiimire, Allyson M. Pollock

**Affiliations:** 1 Population Health Sciences Institute, Newcastle University, Baddiley Clark Building, Richardson Road, Newcastle upon Tyne, NE2 4AX UK; 2 Epidemiology and Public Health, Swiss Tropical and Public Health Institute, Basel, Switzerland; 3 Centre for Trust, Peace and Social Relations, Coventry University, Coventry, UK; 4 Faculty of Medicine, Mbarara University of Science and Technology, Mbarara, Uganda

**Keywords:** Access to essential medicines, Essential medicines list, Registration, Good manufacturing practice, Quality assurance, Uganda, Universal health coverage

## Abstract

**Background:**

Universal access to high quality essential medicines is critical to sustainable development (SDG 3.8). However low- and middle-income countries struggle to ensure access to all medicines on their national essential medicines lists (EML). Market registration is the first step in determining both access and availability yet the extent to which essential medicines are registered for use at country level is not known. Companies apply for a marketing authorisation, however low price or lack of a market is a disincentive. Local production has been promoted to ensure availability of essential medicines but research in this area is also limited.

**Methods:**

The study took place between 2011 and 2015. We systematically examined the registration status of medicines and vaccines listed in the Ugandan 2012 EML and conducted 20 interviews with regulators, ministry of health representatives, donors, and pharmaceutical producers and analysed quality assurance issues affecting registration, procurement, and local production of medicines in Uganda. In 2017 we conducted a further three interviews to clarify issues around non-registration of essential medicines highlighted by our analysis.

**Results:**

Of the 566 essential medicines and vaccines nearly half (49%; 275/566) had no registered product in 2012. Of the 3130 registered products, just over a quarter (28%; 880/3130) were listed on the EML. Six local producers had registered 138 products of which 40 corresponded to 32 unique essential medicines. Interviews highlighted alternative routes to availability other than registration. Local producers faced considerable barriers to achieving international quality standards required for international procurement of medicines for the domestic market.

**Conclusions:**

Monitoring and audit of the registration of essential and non-essential medicines should be a priority nationally and, regionally through harmonisation of registration requirements in the East African Community. National and regional manufacturing plans should consider local production of unregistered essential medicines.

## Introduction

Universal access to essential medicines is necessary to provide for the priority health care needs of the population (Sustainable Development Goal 3.8). Essential medicines *“are intended to be available within the context of functioning health systems at all times in adequate amounts, in the appropriate dosage forms, with assured quality and adequate information, and at a price the individual and the community can afford.”* [1]. The concept of essential medicines is widely accepted and many countries have their own national essential medicines list (EML), yet multiple barriers to universal access persist and more evidence and support are needed for effective implementation of medicine policies in low- and middle-income countries (LMICs) [2].

World Health Organization and Health Action International (WHO/HAI) standardised surveys on availability and affordability of registered medicines show that essential medicines are more available than non-essential medicines in both public and private sectors [3].

An unexplored aspect is the registration of essential medicines. There is anecdotal evidence of non-registration of essential medicines at country level but there has been no research in this area.

Continued support for local production of pharmaceuticals to improve access to medicines has been pledged by five United Nations institutions and the Global Fund [4]. The evidence on their extent and contribution to improved access to medicines, especially in low income countries, is limited [5, 6].

In this case study in Uganda we systematically examined the registration and local production of essential medicines. We undertook qualitative interviews with regulators, ministry of health representatives, donors, and local manufacturers to understand the registration and quality assurance issues for imported and locally produced pharmaceuticals.

### Ugandan context

The National Drug Authority (NDA) of Uganda is mandated by the National Drug Policy and Authority (NDP&A) Act to implement the National Drug Policy “*to ensure the availability, at all times, of essential, efficacious and cost-effective drugs to the entire population of Uganda*” [7]. NDA regulates pharmaceutical products for marketing; controls the quality of medicines used in the country; and licenses local pharmaceutical manufacturers, wholesalers, distributors and pharmacies. Since 2001, the Ministry of Health’s Pharmacy Section has been responsible for the Essential Medicines and Health Supplies List for Uganda (‘Ugandan EML’ hereafter).

Uganda is a member of the East African Community (EAC), which in collaboration with New Partnership for Africa’s Development (NEPAD) launched the EAC Medicines Registration Harmonization (EAC MRH) project in 2012. The project has been supported by the WHO, the Bill & Melinda Gates Foundation and the World Bank [8, 9]. The EAC MRH aims to harmonise technical requirements for quality assurance such as good manufacturing practice (GMP) standards and registration processes. Cooperation between participating national medicines regulatory authorities (NMRAs) is expected to strengthen local regulatory expertise and lower the regulatory workload.

The occurrence of falsified and substandard medicines in the country [10, 11] has been attributed to NDA’s inadequate staffing levels that limit their ability to inspect medicines at border points [12]. Nevertheless, strengthening of medicines regulation in the recent years has been recognised through the international certification of the NDA for its regulatory activities including drug registration, licensing and post-marketing surveillance (ISO 9001:2015) and WHO pre-qualification and ISO accreditation of the National Drug Quality Control Laboratory (NDQCL) for testing of medicines and medical devices [13].

According to the Ministry of Health (MoH), availability of essential medicines and health supplies improved from 43 to 63.8% between 2009/10 and 2014/15 due to increased funding by both government and foreign donors. Foreign funds accounted for more than 70% of public spending on medicines and mainly targeted HIV/AIDS, malaria and TB which in turn accounted for more than half of government spending on essential medicines and supplies [14].

## Data sources and methods

The main study was conducted between 2011 and 2015.

### EML registration status and local producers

EML: The 2012 Ugandan EML is publicly available and lists 566 essential medicines and vaccines [15] as general or specialist medicines, designated for a specific level of the health care system, and classified as vital, essential, or necessary (VEN) to help facilities prioritise medicines in the context of limited budgets (See details in Table 1).

**Table 1:** Categorisation of medicines and vaccines in the Ugandan Essential Medicine List (MoH 2012)

**Level of use** – each item has a specified lowest level of use within the health care system and can be prescribed and dispensed in the specified and all higher levels	
NR National referral hospital	
RL Regional laboratory	
RR Regional referral hospital	
H Hospital	
HC4 Health centre 4 (Medical Officer)	
HC3 Health centre 3 (Clinical Officer)	
HC2 Health centre 2 (Enrolled Comprehensive Nurse)	
HC1 Health centre 1 (Community level)	
**Specialist list** further restricts to facilities with a specific type of clinical and/or diagnostic expertise (e.g., certain ophthalmological preparations)	
**VEN classification** takes into account health impact	
Vital (V)—medicines used to treat life-threatening diseases and health supplies and laboratory commodities that are necessary for basic healthcare	
Essential (E)—medicines are effective to treat less severe, but nevertheless, widespread illnesses	
Necessary (N)—medicines used for diseases with less impact on the population, medicines of doubtful efficacy, or medicines with a high cost for marginal therapeutic benefit	
Items deemed vital for HC4 are assumed to be vital at all higher levels of health system but may not be ordered if other better alternatives are available to the higher level	

The Ugandan EML comprises 5 sections: A) General Medicines, B) General Health Supplies, C) Specialist Medicines, D) Specialist Health Supplies, and E) Laboratory Supplies. We collected information on medicines and vaccines listed in sections A) and C), their specified dosage form and strength, recommended level of use, and VEN classification.

NDR: The National Drug Register of Uganda (NDR) is publicly available and continuously updated. It listed 3130 pharmaceutical products when accessed on 28 November 2012.

We compared the EML against the NDR and recorded the number and names of foreign and local manufacturers of essential medicines (see Additional file 1 for how differences between the EML and NDR listings were resolved). We looked to see which essential medicines had a WHO prequalified manufacturer/product [16, 17] as of November 2012.

### Interviews

Between 2011 and 2015 we conducted semi-structured interviews with 20 key informants recruited from government bodies, professional councils, pharmaceutical producers and distributors, and international organisations (see the list of key informants in Additional file 2). Our interview guides were adapted to a key informant’s role focusing on issues affecting: registration (regulatory capacity of the NDA, funding, alternative routes); local production (GMP compliance and enforcement); procurement (donor support and GMP compliance) (see Additional files 3, 4, 5 and 6 for Interview guides). In 2017 we undertook a further three interviews to clarify issues around non-registration of essential medicines.

Government reports and regulatory guidelines by NDA, WHO and EAC MRH were consulted to gather published information on regulatory procedures and capacities.

Ethical Approval was obtained for this study. Consent, ethics and data storage were discussed prior to the interview. Interviews were transcribed, entered into MAXQDA (version 10) software and coded with reference to study aims. A repository system, Alfresco, was used to anonymously store data securely and an identification number was given.

## Analysis and results

### Registration status of essential medicines

Of the 3130 brands of human medicines and vaccines registered on the 2012 NDR, 880 (28%) corresponded to medicines and vaccines listed on the Ugandan EML. The 2012 version of the EML contained 566 essential medicines and vaccines (unique INN/dosage form/strength); 275 (49%) were not registered with the NDA. Of the 291 (51%) registered medicines, 37 were listed as a different salt, dosage form, or strength but were considered to be reasonable/practical substitutes. Of the 275 essential medicines that were not registered, 166 (60%) were listed as general medicines and 109 (40%) as specialist medicines (Table 2). One quarter (42/166) of the general medicines were classified as vital and included: BCG vaccine, lignocaine, morphine, rifampicin combinations, diphtheria-pertussis-tetanus vaccine, and warfarin.

**Table 2:** Number and percentage of essential medicines not registered with the NDA according to VEN classification and the level of use in the health system

EML section	VEN classification			Level of use		
		EML	Not registered		EML	Not registered
**General medicines**	Vital	135	42 (31%)	HC2 (HC1)	18	7 (39%)
	Essential	143	60 (42%)	HC2	69	23 (33%)
	Necessary	129	64 (50%)	HC3	68	14 (21%)
				HC4	112	45 (40%)
				H	74	39 (53%)
				RR	59	32 (54%)
				NR	7	6 (86%)
	**Subtotal**	**407**	**166 (41%)**	**Subtotal**	**407**	**166 (60%)**
**Specialist medicines**	Vital	48	32 (67%)	HC2 (HC1)	0	–
	Essential	50	32 (64%)	HC2	0	–
	Necessary	61	45 (74%)	HC3	2	1 (50%)
				HC4	13	6 (46%)
				H	19	12 (63%)
				RR	72	49 (68%)
				NR	53	41 (77%)
	**Subtotal**	**159**	**109 (69%)**	**Subtotal**	**159**	**109 (69%)**
**Total**		**566**	**275 (49%)**		**566**	**275 (49%)**

1. HC health centres level 1–4, H hospital, RR regional referral hospital, NR national referral hospital

2. For medicines listed more than once and with a different VEN classification and/or level of use we considered the highest priority of VEN classification and the lowest level of use. E.g. fluorouracil injection 50 mg/ml was listed as necessary (N) at regional referral hospital level (RR) in the anti-metabolites section of General Medicines and as vital (V) at national referral hospital level (NR) in the cytotoxic section of Specialist Medicines. For the purposes of this table was fluorouracil counted in as a general medicine, V and RR level

Specialist medicines were less likely than general medicines to have a registered product; and medicines listed for hospital levels (H, RR, NR) were less likely to be registered than those for health centres level. In the general medicines category, vital items were more likely to be registered than essential items, and these in turn were more likely to be registered than necessary items (Table 2).

### Regulatory registration shortcuts

Because of lack of regulatory capacity in the national regulatory authorities of LMICs WHO has recommended using approval decisions of well-established regulatory authorities (formerly known as stringent regulatory authorities) and WHO prequalified products [17–19]. The EAC harmonisation project that introduced joint inspections of production facilities and joint registration of medicines for the common EAC market is also intended to reduce workload [20].

**Fig. 1:**
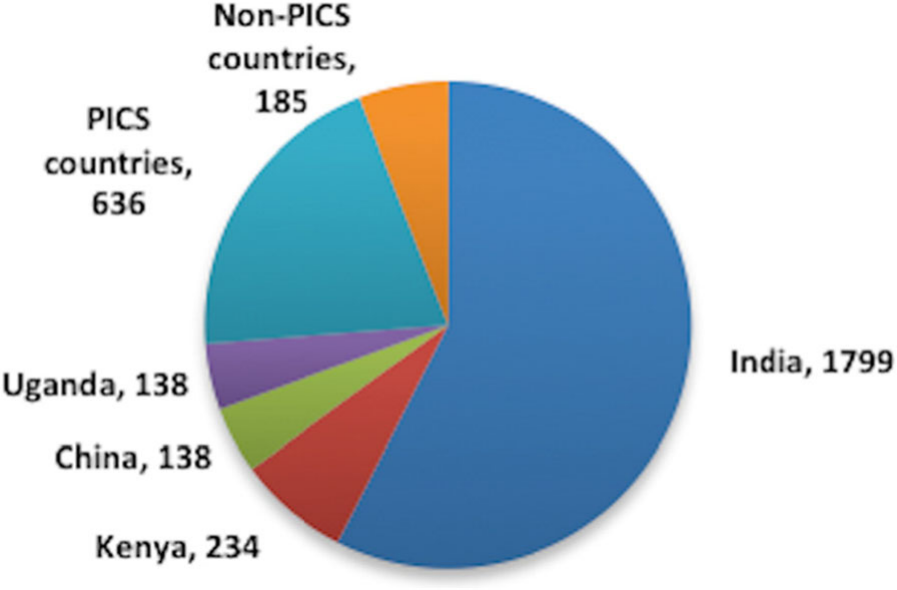
Countries of origin for medicines on the Ugandan National Drug Register, 2012. PIC/S countries: Germany, UK, Cyprus, South Africa, Belgium, France, Malaysia, Switzerland, Indonesia, Italy, Sweden, Canada, The Netherlands, Greece, Spain, USA, Portugal, Denmark, Hungary, Slovenia, Korea, Finland, and Japan. Non-PIC/S countries (importing < 4% of products into Uganda): Pakistan, Egypt, Jordan, Morocco, Turkey, Bangladesh, Iran, and UAE

Uganda imported 96% (2992/3130) pharmaceuticals on its 2012 NDR. Sixty per cent (*n* = 1799) were from India; about 20% of registered products were manufactured in countries with well-established authorities or members of Pharmaceutical Inspection Cooperation/Scheme (PIC/S) (Fig. 1).

Previously all manufacturers irrespective of location were inspected (1-Regulatory 2011). Now under new guidelines introduced in 2016, applications from manufacturers located in countries with a well-established authority undergo a document review (2-Regulatory 2017). If this policy had applied in 2012 it would have reduced workload by no more than 20%, as a high proportion of products are imported from India and other non-PIC/S countries (i.e. countries without well-established authorities).

In 2013 WHO introduced a collaborative registration procedure, which allows the participating NMRAs to take advantage of assessments completed through the WHO prequalification and accelerates registration of prequalified medicines and vaccines at the national level. Uganda, being one of the participating NMRAs, registered ethinylestradiol + levonorgestrel tablets 30 μg + 150 μg by Famy Care Ltd. through this procedure in May 2013.

WHO prequalification does not cover all essential medicines. The WHO prequalification covers medicines in seven therapeutic areas - HIV/AIDS, Malaria, TB, Reproductive Health, Influenza, Diarrhoea, and Neglected Tropical Diseases – and vaccines. Eight per cent (22/275) of the essential medicines without a registration in Uganda had a WHO prequalified manufacturer/product.

### Alternatives to registration: special import licences

A special import licence can be given for medicines not listed on the national drug register (8-Regulatory 2011, 19-Distribution 2015). The NDP&A Act allows for medicines to be authorised and imported in emergency or extraordinary circumstances [7]. *“[U]sually these applicants are programmes that are implemented by the MoH [with] donations from international agencies like UNICEF, UNFPA, Global Fund, WHO, and USAID. [...] There are also pharmacies registered with NDA that bring certain drugs, not registered, mainly anti-cancer medicines”* (2-Regulatory 2017).

These applications undergo a document review in which WHO prequalification or a registration and a GMP certification issued by a well-established authority are confirmed (2-Regulatory 2017; 22-Donors/NGOs 2017)*.* Such applications are common. *“Every week we get applications for unregistered drugs from these programmes. [In 2016], NDA received 660 applications for special import of unregistered drugs out of total of 7617 applications for verification, which is about nine per cent”* (2-Regulatory 2017).

According to a Medecins Sans Frontieres (MSF) pharmacist MSF supplies are centralised through three European centres that identify sources and check compliance with stringent quality standards.

*“Supply centres export to all countries, so it is difficult to comply with all the regulations. If there are two choices and one source is registered […], then the supply centre would choose the registered one. […] We import registered and non-registered medicines, knowing that non-registered [ones] take longer. Especially if we want to import non-registered product with several alternatives in the local market, we have to negotiate with the NDA”* (22-Donors/NGOs 2017).

### Local production of essential medicines

In 2011 there were 14 licensed pharmaceutical producers in Uganda [21]. Local manufacturers focused on the formulation of finished pharmaceutical products and repackaging; no active pharmaceutical ingredients were produced locally. Six local manufacturers had products listed on the 2012 NDR; 40 of their 138 products (29%) corresponded to 32 unique medicines listed on the Ugandan EML (Table 3).

**Table 3:** Local production of essential medicines in Uganda

Manufacturer	Number of NDA registered products	Number of products corresponding to EMs
ABACUS	9	6
KPI	57	16
MEDIPHARM	14	0
QUALITY CHEMICALS	1	1
RENE INDUSTRIES	55	17
SEV PHARMACEUTICALS	2	0
Total	138	40

Source: Our analysis of NDR (2012) and Ugandan EML (2012)

In 2010 it was reported that local producers in Uganda met around 10% of local essential medicine demand [22]. To support local industry the government introduced reduced import tariffs and price preference policy of 15% favouring the local producers in 2012 [23]. Later assessments, however, noted insufficient impact of the price preference policy [24] as domestic producers were often unable to meet the demand [25].

Our interviews with local producers highlighted two challenges – stringent international production standards such as GMP, and funding. Compliance with GMP was accepted as compulsory and the NDA highly respected (10- and 12-Manufacturing 2013). However, manufacturers felt pressured to keep pace with the international GMP standards (10- and 11-Manufacturing 2013). Only one manufacturer, Cipla Quality Chemical Industries Limited (CQCI), gained a WHO prequalification as an alternative manufacturing site for Cipla’s (India) lamivudine, nevirapine and zidovudine fixed dose tablet preparations.

### Incentives for production and registration of essential medicines

The second national pharmaceutical sector strategic plan (2010/11–2014/15) emphasized the importance of increased funding for essential medicines [26] which in turn creates demand for these products.

#### Selection and procurement of medicines

The public procurer, National Medical Stores (NMS), procure medicines and health supplies according to the EML listing and special requests by MoH.

Key Informants responsible for procurement and distribution of medicines reported limiting supplies to products registered with the NDA (14-, 16- and 18-Distribution 2011–2012). All of them emphasized the need for internal quality control in storage and distribution after they receive a consignment (13- to 18-Distribution 2011–12). With limited budgets procurement of essential medicines was further prioritised using the VEN (vital, essential, necessary) classification (13-Distribution 2011; 19-Distribution 2015). If the likelihood of public procurement was low and products were not bought in the private sector, products were unlikely to be registered (8-Regulatory 2011; 16-Distribution 2011).

#### Donors procurement policies

International procurement policies of donors and NGOs disadvantage local producers if they have neither WHO prequalification nor an approval from a well-established authority. One local manufacturer gave the example of a UN agency visiting their facility, excited about a product, but who immediately disengaged upon realising the facility was not WHO prequalified. Despite the manufacturer demonstrating the technical quality and capabilities of their facility, and investments that had been made to increase quality control, the manufacturer’s lack of WHO prequalification was a barrier to the UN agency procuring medicines from them (10-Manufacturing 2013).

Until 2014, MSF procured locally but since then the main policy has been importation. The key reasons given for this change were low production capacity and quality standards of the local pharmaceutical industry. Current MSF procurement relies on WHO prequalification but will not recognise GMP certification by the Ugandan NDA. (22-Donors/NGOs 2017). As a result WHO prequalified Cipla Quality Chemical Industries Limited (CQCI) is the only local manufacturer they can procure from in Uganda.

#### EAC joint registration of medicines

Joint registration for the EAC common market could motivate manufacturers to produce and register essential medicines. Although one manufacturer estimated that registration time increased from 3 to 6 months to one year and noted that the assessment was becoming more stringent (10-Manufacturing 2013), this could be outweighed by the automatic access to a larger market:

*“[I]f you register with Uganda, at the moment, you also pay when you register with Kenya and Tanzania. We have to pay for a site visit and registration of each product, … a renewal fee. … But [with] this harmonisation, […] you can choose any one country for registration. […] So once the NDA says the product is approved, I can automatically supply to [the other EAC countries]”* (12-Manufacturing 2013).

Specific strategies to promote local manufacturing of essential medicines are formulated in the third national pharmaceutical sector strategic plan published in 2015 They include tax incentives and subsidies, encouraging local and international procurers to procure locally produced essential medicines, support for achieving additional regulatory certification, e.g. WHO prequalification, and establish “mechanisms to allow for harmonisation of policies and their reciprocity on domestic pharmaceutical manufacturing among EAC countries” [27]. At the regulatory level, however, apart from programme medicines for the MoH, no priority is given to essential medicines (2-Regulatory 2017).

## Discussion and conclusion

Marketing authorisation is the first step in determining access and availability of medicines. In 1985 the Nairobi Conference for rational medicines use policy advocated that registration should be limited to the essential medicine list and prioritised at the regulatory level [2]. In practice this has never been implemented.

More than 70% of products on the Ugandan 2012 NDR were non-essential. This has the effect of using scarce regulatory resources and enabling the private market where patients pay out-of-pocket. Regulatory resources could be used more effectively if essential medicines are prioritised. Use of restricted lists of medicines also improves prescribing practices and contributes to appropriate use of medicines.

In Uganda almost half of essential medicines had no registered product with the Ugandan regulatory authority in 2012. While unregistered medicines and vaccines can be imported on a special permit granted by the NDA, this channel is generally slow, and is designed for exceptional circumstances and emergency situations. Although availability of essential medicines without registration was reported in private pharmacies and drug shops [28], these products avoided regulatory channels and scrutiny required for quality assurance. Our findings show that essential medicines prioritised for public procurement through the VEN classification are more likely to be registered at the country level thus highlighting the importance of funding for all essential medicines identified for universal health coverage target. Although the government funding has been increasing more than half of funds is spent on three therapeutic areas – HIV/AID, malaria, and TB. Overall government per capita spending on Ugandan EML products was about US$ 2.4 in 2013/14, significantly short of the estimated requirement of US$ 12 [14]. Out-of-pocket expenditures, primarily spent on medicines in the private market, remained high at about 40% of total health expenditure [27].

Research has shown that registration fees are not a barrier to registration in general [29, 30]. However South African producers reported registration costs, GMP inspection fees and inspections as reasons for not exporting medicines to other African countries including Uganda [31].

Where NGOs purchase high-volume priority medicines, procurement and associated enforcement of GMP requirements and dossier assessment are at the international level. International manufacturers may then bypass registration with national medicine regulatory agencies. This in turn affects interest of local manufacturers in producing essential medicines: if local producers are unable to meet international standards they cannot supply their products to UN and NGO programmes even where the programmes are in country.

To our knowledge this is the first study looking at the registration of an entire country list of essential medicines. The methodology used in this study could be applied to all low- and middle-income countries to audit essential medicine registration and to highlight priorities for registration. In 2018 the national regulatory authority of Ethiopia announced they would expedite the registration process for highly important medicines needed in the country [32] signalling that the non-registration of essential medicines is not unique to Uganda of 2012.

### Recommendations

Essential medicines should be prioritised by the regulatory system and continuous monitoring of registration of essential medicines is necessary.

Uganda’s pharmaceutical sector development plan should address the issue of unregistered essential medicines and the implications for availability and use.

Joint inspections, audits and registrations with other EAC countries and prioritisation of essential medicines could enhance availability. Further work is needed to identify strategies to incentivise production and registration of essential medicines for the common EAC market.

## Supplementary information

**Additional file 1.** Resolution of differences between the Ugandan EML and NDR.

**Additional file 2.** Key informants.

**Additional file 3.** Interview guide regulators.

**Additional file 4.** Interview guide procurers/distributors.

**Additional file 5.** Interview guide donors/NGOs.

**Additional file 6.** Interview guide regulators follow up.

## Data Availability

All data used in quantitative analysis were sourced from publicly available sources and are available from the corresponding author on reasonable request. Full transcripts arising from this study are not publicly available due to concerns about identifying participants. Participants of this study did not provide consent for transcripts to be publicly shared or used in another project. We are following our ethics procedures for the AMASA project, for which institutional ethical approval was given. For the purposes of validating and verifying our data requests by qualified researchers may be directed to the corresponding author.

## Abbreviations

CQCI: Cipla Quality Chemical Industries Limited
EAC: East African Community
EAC MRH: East African Community Medicines Registration Harmonization
EML: Essential Medicine List
GMP: Good Manufacturing Practice
H: Hospital
HC1–4: Health centre 1–4
INN: international non-proprietary name
LMICs: Low- and middle-income countries
MoH: Ministry of Health
MSF: Medicins Sans Frontiers
NDA: National Drug Authority
NDP&A: National Drug Policy and Authority
NDQCL: National Drug Quality Control Laboratory
NDR: National Drug Register
NEPAD: New Partnership for Africa’s Development
NMRAs: National Medicines Regulatory Authorities
NMS: National Medical Stores
NR: National referral hospital
PIC/S: Pharmaceutical Inspection Cooperation/Scheme
PSU: Pharmaceutical Society of Uganda
RL: Regional laboratory
RR: Regional referral hospital
VEN: vital, essential, necessary
WHO/HAI: World Health Organization / Health Action International
