# Supplementary material for: Registration and local production of essential medicines in Uganda
Source: J Pharm Policy Pract. 2020 Aug 11;13:31. doi: 10.1186/s40545-020-00234-2 (PMC7419186; doi:10.1186/s40545-020-00234-2)
Supplement: Supplementary file 1 — Additional file 1. Resolution of differences between the Ugandan EML and NDR. [file 40545_2020_234_MOESM1_ESM.docx]

**Additional file 1. Resolution of differences between the Ugandan EML and NDR**

| **Differences** | **Solution** |
| --- | --- |
| 1. Typos in the medicine name | The EML and NDR list were checked by at least two researchers to ensure that we did not miss any relevant products and their manufacturers |
| 1. Different strength | 1. The strength on the EML is higher than on the NDR and use of multiple doses equals to strength recommended by the EML-> list the product available on the NDR with a note about the licensed strength 2. The strength on the EML is lower than on the NDR -> no registered product corresponding to the essential medicine |
| 1. Tablet vs. capsule | Although the tablet and capsule formulation are pharmacologically different we assumed that they can be used interchangeably in practice |
| 1. Tablet vs. uncoated tablet vs. tablet FC (film coated) | Can be used interchangeably in practice |
| 1. Injection vs. infusion | Not the same and cannot be used interchangeably |
| 1. Solution vs. cream | Not the same |
| 1. Injection vs. powder for injection vs. solution for injection | Considered to be interchangeable |
| 1. Powder for injection vs. lyophilized injection | The same |
| 1. Suspension vs. syrup | Decided on case by case basis |
| 1. Medicine salts | Decided on case by case basis  If the exact INN/dosage form/strength available -> excluded salts  If the exact INN/dosage form/strength not available > recorded available salt products |
