# Supplementary material for: Registration and local production of essential medicines in Uganda
Source: J Pharm Policy Pract. 2020 Aug 11;13:31. doi: 10.1186/s40545-020-00234-2 (PMC7419186; doi:10.1186/s40545-020-00234-2)
Supplement: Supplementary file 2 — Additional file 2. Key informants. [file 40545_2020_234_MOESM2_ESM.docx]

**Additional file 2. Key informants**

**Regulatory (1-8):** NDA (5); MoH, pharmaceutical division (1); Medical and dental practitioners council (1); Pharmaceutical Society Uganda (1)

**Manufacturing (9-12):** executive officer (1); commercial officer (1); quality assurance pharmacist (2)

**Distribution (13-19):** public procurement and distribution (3); private non-profit procurement and distribution (2); private for-profit procurement and distribution (2)

**Donors/NGOs (20-23):** donor (1); international organisations (2); local NGO (1)
