# Supplementary material for: Registration and local production of essential medicines in Uganda
Source: J Pharm Policy Pract. 2020 Aug 11;13:31. doi: 10.1186/s40545-020-00234-2 (PMC7419186; doi:10.1186/s40545-020-00234-2)
Supplement: Supplementary file 3 — Additional file 3. Interview guide regulators. [file 40545_2020_234_MOESM3_ESM.docx]

**Additional file 3. Interview guide regulators**

**Questions focusing on general interests and particular tracer medicines**

1. **Staff, experience and turnover:**

What is the capacity of your agency to control the medical products in your markets?

- Do you have sufficient staff? If not, what could be dome to change this? In what specific functions? Describe the process for appointing staff & any associated issues.
- What is the level of staff experience? Is adequate training/capacity building available for staff?
- What is the frequency of turnover and ability to retain good staff (retention)? What could be the reasons for turnover? What is necessary to retain good staff?
- How is the workload managed? Is enough priority given with regards to specific therapeutic areas e.g. tracer medicines (TMs), small & large scale producers of TMs?
- How are priorities set within the agency especially with regards to management of critical issues and their impact on the organization?

1. **Financial resources:**

What are the financial resources available to support regulatory work in your country/region?

- Could you clarify the fee structure for services performed by regulatory officials including funding allocation & disbursement procedures: Please comment on:
  - Product dossier evaluation
  - Laboratory support
  - Pharmacovigilance
  - Enforcement activities & whether they are dependant upon external funding (World Bank, WHO, etc.)
  - Surveys/information collection
- Which activities would you find most under-resourced?
- Is there a budget dedicated for office material?
- Is there access to telephones, computers, fax, copiers?

1. **Drug Product review activities:**

What are the main challenges associated with drug product review activities especially with regards to TMs?

- What is the procedure for drug product reviews at the agency?
- Please comment as to the rigor of the registration procedure with respect to TMs.
- Does regulatory practice differ for the public vs. private sectors?
- Are guidelines with regards to TMs available & are they adhered to?
- Are there any difficulties maintaining a data base for tracking activities?
- Please comment on the resources with regards to qualified personnel.
- Are there competing resources for other product categories (control of traditional & herbal medicines, OTCs)?
- Are sampling and testing medicinal products for quality control causing difficulties for the agency and if so, please explain.
- Please describe the scope of activities at various stages of supply chain.
- Is there a post-authorization framework which covers the processing of variations (i.e. manufacturing changes) as well as fast track procedure for urgent safety changes and an effective PV system?
- Is there an effective system to address critical issues that may occur?

1. **Good Manufacturing Practices:**

Can the availability of data from GMP inspections including the frequency be clarified especially with reference to TMs?

- Who has access to inspection reports and how is the data is made available, if at all, to other concerned parties (other regulators & agencies, others)?
- Do you collaborate with other regulatory authorities with regards to conducting inspections?
- Describe the archiving process for the collected data (quarterly, yearly, by region/entire country).
- Are there any differences with regards to inspection frequencies of large and small scale producers & describe any issues related to these.
- Are there enough resources to perform inspections of the necessary facilities?
- Are there any data with regards to GMP inspections of manufacturing sites producing TMs?
- Are overseas GMP inspections conducted with regards to TMs?
- What is the procedure for the enforcement of non-compliance to GMP & how are legal actions taken?
- What are the differences in GMP inspections conducted with regards to the public vs. private sectors?

1. **Good Distribution Practices:**

Can the availability of data from GDP inspections including the frequency be clarified especially with reference to TMs?

- Who has access to inspection reports and how is the data is made available, if at all, to other concerned parties (other regulators & agencies, others)?
- What GDP guidelines are currently being followed?
- At what points of the distribution network are the inspections conducted & how rigorous are the inspections?
- Who are conducting the inspections and are any performed on a voluntary basis?
- Describe the archiving process for the collected data (quarterly, yearly, by region/entire country).
- What is the procedure for the enforcement of non-compliance to GDP & how are legal actions taken?
- Is there support from local law enforcement agencies with regards to GDP and does political will exist?
- What are the challenges for conducting GDP inspections (vast geographic areas, inaccessible locations, other limitations)?
- Are there any specific issues with regards to GDP public sector facilities including frequency, availability of reports, inspecting officials, adequacy of inspections, handling of non-compliance issues, specific issues with regards to TMs?
- How are the GDP inspections of distribution channels under the national disease control programs functioning especially for the TMs with regards to frequency, inspecting officials, availability of data, handling of non-compliance issues?

1. **Harmonization Activities:**

What is the relationship to other regulatory agencies at all levels (e.g., inspections, sharing information, and harmonization of regulations)?

- Describe the collaboration with global regulatory initiatives (ICH) and regulatory bodies (WHO).
- Describe the collaboration with other stringent regulatory authorities (EMA, USFDA, Swissmedic, Health Canada, etc.).
- Is the WHO Certification scheme used? Are there any limitations?
- How do harmonization activities influence the development of guidelines, capacity building initiatives?
- Are there limitations to harmonization efforts such as feasibility for local settings, additional financing and staff, government procurement issues, effects on small domestic producers.

1. **Advertising and Promotion:**

How will the promotion of products generally and with reference to tracer medicines be regulated and what are the challenges associated with this?

- Which unit is responsible for the activity and what is the extent of its authority; are any other parties involved and if so, how is this coordinated?
- Is there adequate financial capacity and technical expertise available for this function?
- Does data exist on non compliance or unethical drug promotion and associated legal actions and where can this data be obtained?
- Are there any major issues confronting the regulators such as lobbying by various groups?
- What are the measures in place to control unethical promotion of TMs and is enforcement effective?
- Are any NGOs or other groups supporting this activity?

1. **Regulations:**

How have regulations changed/evolved over the past decade or two and what has been the impact?

- Please describe any new or pending regulations and the impact on public health.
- Please describe the major policy makers and how they support or oppose new or pending regulations.
- How would you describe the transparency of the regulatory procedures in place?
- Is your regulatory system using up-to-date computerization techniques and what will be the implications on the effectiveness of the drug regulation process?
- Are there any special courts that deal with pharmaceutical related issues?

1. **Pharmacovigilance Program:**

How is your current pharmacovigilance program functioning and what are the main challenges encountered?

- How well is the PV policy implemented in practice?
- Are there any data specific to the TMS?
- How are the data being reported?
- Are medical professionals aware of the reporting system and is it being used adequately?
- Are there any funding issues?
- Are there differences with regards t reporting in the public vs. private sectors?
- Are there current plans to strengthen the existing PV system?
- Are risk management plans a regulatory requirement and for which drugs?

1. **Counterfeit Drugs:**

How are counterfeit, fake, and substandard medicines addressed within the regulatory framework?

- Are the issues around counterfeits and substandard medicines a priority within the agency?
- Please describe the policies and operations association with them.
- How are the counterfeit medicines identified?
- What are the market statistics for counterfeit medicines in general and with regards to the TMs?
- What is the definition of “counterfeit medicine” within the agency and clarify the impact it may have on access to affordable medicines (particularly generics) with respect to TMs
- Is the definition of counterfeit medicine aligned with that of WHO IMPACT?
- What legislation is in place to combat counterfeit medicines and how is it enforced?
- Can some examples be provided of cases involving counterfeit medicines and how they are handled by the local law enforcement agency and at the regional and international levels.
