# Supplementary material for: Registration and local production of essential medicines in Uganda
Source: J Pharm Policy Pract. 2020 Aug 11;13:31. doi: 10.1186/s40545-020-00234-2 (PMC7419186; doi:10.1186/s40545-020-00234-2)
Supplement: Supplementary file 4 — Additional file 4. Interview guide procurers/distributors. [file 40545_2020_234_MOESM4_ESM.docx]

**Additional file 4.** **Interview guide procurers/distributors**

**Organization and staffing (1)**

Can you comment on the organizational personnel structure of this unit?

What are the qualifications of the other staff?

Do the staffs have any form of continuous training in stores management?

Are there guidelines to guide staff in supply management?

What other challenges do you face with your personnel?

Are there policies or restrictions that in your opinion are limiting or enabling access to medicines?

How would you summarize access to medicines at this facility?

**Logistics management information system (2)**

Do you have any logistics management information system?

What logistics data do you usually record?

With which other institutions or organizations do you usually share your logistics data with?

Have those interactions been beneficial to you?

**Forecasting (3)**

How is the AMC computed?

For our study medicines have you ever had any challenges forecasting their quantities?

**Obtaining supplies/procurement (4)**

Apart from you who else is on the committee for procurement planning and forecasting?

How is the process coordinated?

What logistical element does the procurement process take into account?

When you make orders, it is specifically to JMS and NMS or are there other sources?

Who authorizes the suppliers?

Do you consider the essential drugs list when forecasting?

When you make forecasts, are the amounts accurate or do you have under or over estimations?

The pipeline through which your drugs pass, is it regularly monitored so as to prevent stock outs?

Any comments or suggestions that can be done to improve procurement?

**Inventory control procedures (5)**

What types of inventory control systems do you have?

What information is recorded in the dispensing log?

From NMS to you it is the pull system, how about from here to the various units?

What kind of feedback do you get from the ward units?

How do you handle stock imbalances like overstock or under stock?

How do you handle expired medicines?

Have you found any challenges in handling expired medicines?

Apart from stock cards are there any other systems for tracking losses and other adjustments that occur in the stock?

How do you get to know that such a thing has happened?

For drugs like coartem, Lamivudine, metformin, Oxytocin and Rifampicin, have you had any stock outs in the last one year?

How long do the stock outs usually take?

In what ways have the stock outs affected you and your work?

What suggestions can make that can be done to improve inventory control?

**Warehousing and storage (6)**

How often are the checks on inventory done?

How do you do the physical counts?

Is the storage space adequate to handle the current quantities of drugs you have?

Are there any improvements that you would want to be done in the storage of drugs?

Are there practices to ensure quality of drugs at the different stages they are handled when they are in the hospital?

**Transportation and Distribution (7)**

To whom do you distribute?

How are medicines distributed from the distributor’s level to the service delivery level?

What challenges have you faced in the delivery of your medicines?

**Organizational support for logistics system (8)**

What is the procedure for supervisory visits?

Do you conduct supervisory visits? Are supervisory visits conducted on you?

Have the supervisions been beneficial to you? How have you used it?

Do the staffs involved in logistics have enough tools and resources to do their jobs?

Do you have any comment on the strengths or weaknesses of the support for the logistics system of the hospital?
