# Supplementary material for: Registration and local production of essential medicines in Uganda
Source: J Pharm Policy Pract. 2020 Aug 11;13:31. doi: 10.1186/s40545-020-00234-2 (PMC7419186; doi:10.1186/s40545-020-00234-2)
Supplement: Supplementary file 5 — Additional file 5. Interview guide donors/NGOs. [file 40545_2020_234_MOESM5_ESM.docx]

**Additional file 5. Interview guide donors/NGOs**

**Questions on medicines quality assurance, procurement and import**

Do they procure medicines outside Uganda only, or in Uganda, or both?

Do they procure and import into Uganda only medicines registered with the Ugandan NDA?

Do they procure only essential medicines listed on the Ugandan essential medicines list (EMSHLU)? Or do they have their own formulary?

Who is responsible for GMP checks, i.e. who checks that the supplying manufacturers comply with GMP standards?

Are GMP inspections conducted by the procurer or do they rely on GMP certificates issued by regulatory authorities? If they rely on GMP certificates, do they accept a certificate by any national regulatory authority, or only US FDA or EMA, PICs countries, WHO prequalification, etc.?

If importing into Uganda medicines not registered by the Ugandan NDA do they need to request the NDA to issue a special permit (to import the medicines)?

What is the process when procuring medicines not registered with the NDA?

            Who is responsible for quality assurance?

Who checks for GMP compliance?

Are in such cases GMP inspections conducted or GMP certificates checked? Is there a different treatment in case of a WHO prequalified manufacturer or a manufacturer with a GMP certificate issued by a stringent regulatory authority versus Indian manufacturers?

Are the samples of imported medicines tested systematically by the NDA for quality?

What proportion/how many products (not registered with the NDA) do they procure annually? Are these typically related to certain health issues or low volume medicines?

To whom do they supply? Only their own programmes (what programmes/projects do they have in Uganda?) or also to other private providers, pharmacies?
