# Supplementary material for: Registration and local production of essential medicines in Uganda
Source: J Pharm Policy Pract. 2020 Aug 11;13:31. doi: 10.1186/s40545-020-00234-2 (PMC7419186; doi:10.1186/s40545-020-00234-2)
Supplement: Supplementary file 6 — Additional file 6. Interview guide regulators follow up. [file 40545_2020_234_MOESM6_ESM.docx]

**Additional file 6. Interview guide regulators follow up**

Questions focusing on import of non-registered medicines and their quality assurance

1. Who checks for the GMP compliance in cases when the special import permit is granted and what is the process?

2. Are the production sites inspected for the purpose of granting the special permit or reliance on GMP certificates? (if yes, then which certificates are accepted - issued by stringent regulatory authorities, WHO prequalification, other?

3. How many special permits granted on annual basis?

4. Besides NMS, do other procurers (such as JMS) and NGOs (e.g. MSF, Global Fund local agents) also request for special permits to be granted for their procurement?

5. Does the NDA have a list of licensed procurers, importers, distributors and wholesalers?

6. Are the samples checked for quality?
